# The emerging role of the long non-coding RNA *HOTAIR* in breast cancer development and treatment

**DOI:** 10.1186/s12967-020-02320-0

**Published:** 2020-04-03

**Authors:** Hossein Mozdarani, Vahid Ezzatizadeh, Roghayeh Rahbar Parvaneh

**Affiliations:** Department of Medical Genetics, Faculty of Medical Sciences, Tarbiat Modares University, Tehran, Iran

**Keywords:** *HOTAIR*, lncRNA, Normal development, Breast cancer, Therapeutic approaches

## Abstract

Despite considering vast majority of the transcribed molecules as merely noise RNA in the last decades, recent advances in the field of molecular biology revealed the mysterious role of long non-coding RNAs (lncRNAs), as a massive part of functional non-protein-coding RNAs. As a crucial lncRNA, HOX antisense intergenic RNA (*HOTAIR*) has been shown to participate in different processes of normal cell development. Aberrant overexpression of this lncRNA contributes to breast cancer progression, through different molecular mechanisms. In this review, we briefly discuss the structure of *HOTAIR* in the context of genome and impact of this lncRNA on normal human development. We subsequently summarize the potential role of *HOTAIR* overexpression on different processes of breast cancer development. Ultimately, the relationship of this lncRNA with different therapeutic approaches is discussed.

## Background

Investigating human biological system raises the question whether the limited number of genes, in the context of “central dogma of biology” hypothesis, could be the absolute cause of physiological and developmental complexity of human cells? Whilst almost 70–90% of the genomic DNA is estimated to be transcribed, only less than 2% of the genomic DNA is translated to proteins (reviewed by [1]). This implicates the fundamental role of non-coding RNAs (ncRNAs) in the human cell development and survival. In terms of size, ncRNAs are categorized in two classes: small non-coding RNAs and long non-coding RNAs [2]. The length of long non-coding RNAs (lncRNAs) is generally more than 200 nucleotides and they are usually transcribed by RNA polymerase II. ENCODE project estimates more than 28,000 lncRNAs encoded from human genome [3]. Although the function of several lncRNAs is yet undetermined, data show that many recognized lncRNAs contribute to diverse molecular mechanisms in the cells, such as gene methylation and histone modification [4, 5], DNA repair (reviewed by [6]), telomere length (reviewed by [7]), gene regulation (reviewed by [8]), cell cycle progression/arrest [9, 10] and cell differentiation [11]. Misregulation of lncRNAs could cause different abnormalities, including cancers [12]. As an example, the critical role of lncRNA MEG3 have been demonstrated to likely be mediated by epithelia-mesenchymal transition (EMT) in breast, liver, glioma, gastrointestinal, lung malignancies (reviewed by [13]). Among all other malignancies, development of breast cancer [14], as a leading cause of malignancy and mortality in women, worldwide [15] is influenced by many types of lncRNA. In this regards, several lncRNAs have been demonstrated to play oncogenic role in the malignant cells leading to tumourigenesis by participating in diverse processes including cell growth, proliferation, invasion, EMT and metastasis (reviewed by [16]). Within the last decade, *HOTAIR* has been introduced as a crucial oncogenic lncRNA contributing to different processes of breast cancer cell malignancy. Thus, navigating the functions and mechanisms of this lncRNA could further help find novel strategies to prevent or treat this malignancy.

In this review, we aim to briefly outlook the role of *HOTAIR* in breast cancer progression, as a new potential diagnostic and prognostic biomarker. For that, the structure of this lncRNA is generally described followed by highlighting its potential role in normal prenatal and postnatal developments. We next implicate the molecular function of *HOTAIR* in various processes of breast tumourigenesis. Ultimately, the relationship of *HOTAIR* to different therapeutic agents is discussed.

### *HOTAIR* structure

**Fig. 1:**
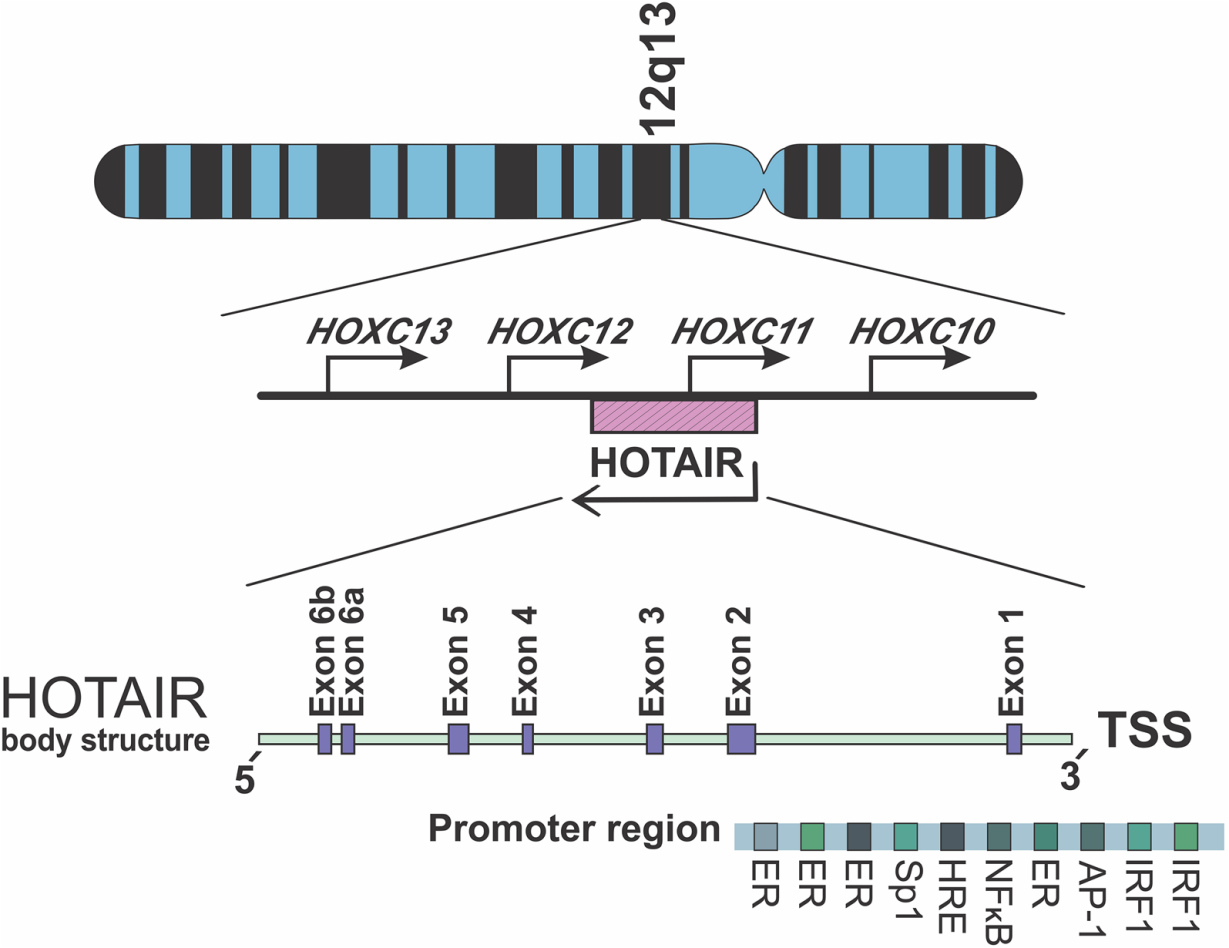
Schematic location of *HOTAIR*. This lincRNA has been located at 12q13.13, between *HOXC11* and *HOXC12* genes, in the antisense strand. It contains six exons (including two domains in the exon 6). The promoter region of *HOTAIR* contains different binding factor location, including ER, IRF1 and NF-κB

In 2007, Rinn and colleagues discovered the lncRNA named *HOTAIR*, by using tailing array of *HOXC* gene locus. This molecule belongs to the long intergenic non-coding RNA (lincRNA) subclass and contains 2158 nucleotides and in human is located on chromosome 12q13.13, between *HOXC11* and *HOXC12* genes [17]. In human, it is only transcribed from antisense strand of the *HOXC* genes and partly overlaps with *HOXC11* (Fig. 1). Despite the fact that nascent forms of this transcript could be spliced, capped and polyadenylated using RNA polymerase II, they do not generate any functional protein [17]. *HOTAIR* has been manifested as of the first lincRNA with trans-binding regulatory capability, contributing to regulation of the distant genes. Evolutionarily, transcription of *HOTAIR* has only been determined in mammalians, including all vertebrates [18].

In contrast to previous reports the mature transcript has recently been affiliated to almost 2.4 kb sequence length (https://www.ncbi.nlm.nih.gov/gene?Db=gene&Cmd=DetailsSearch&Term=100124700; 12 August 2018). Apart from the last exon bearing 1816 nucleotides length, the other exons carry short sequences. Despite several studies indicating that human *HOTAIR* is composed of six exons, recent data analyses introduce it with seven exons, while the last two exons are fairly contiguous to each other (Fig. 1); so that, they have been introduced as two domains of the exon 6 [19].

In terms of transcription, at least five different variants have thus far been detected which might be caused by different factors, like mode of alternative splicing in the related nascent RNA [20]. In addition, at least two alternative promoters have been reported, associating with expression of the *HOTAIR* in different human cells [21]. The principle differences of these transcript variants, in terms of expression level and function, are not yet quite clear. It has also been indicated that 18 enhancers contribute to the regulation of *HOTAIR* expression level [22].

In the genome context, secondary structure of the *HOTAIR* gene body (including exonic and intronic regions), not only coordinates in the establishment of different transcription variants, but also associates with regulation of *HOTAIR* expression levels. In addition to the body structure, flanking regions of this lincRNA might also contribute to the regulation of *HOTAIR* expression. For instance, as a suppressor protein, interferon regulatory factor 1 (IRF1) could bind into the related binding motifs of *HOTAIR* promoter at two positions of 53–64 and 136–148 bp (Fig. 1), upstream of transcription start site [23]. Lu and colleagues also showed that activating DNA methylation of a downstream intergenic CpG island -located between *HOTAIR* and *HOXC12* gene- could alter transcription level of this lincRNA [24]. In silico analyses suggest that most of CpG islands overlap with the active promoter regions, among which there are several DNase I hypersensitive hotspots in some cell lines. Several tandem repeats and single nucleotide polymorphisms (SNP) have also been proposed within the regulatory sequence of this lincRNA [21]. Consistently, in vitro and in vivo studies have demonstrated the role some SNPs in regulation of *HOTAIR* expression level. Thus, rs920778 and rs12826786 polymorphisms correlate with *HOTAIR* up-regulation [25–27]. Considering the impact of some *HOTAIR* SNPs on elevating the corresponding transcription level and consequently cancer susceptibility, evidences suggest that it can be used as a predictive marker in evaluating risk of breast cancer [28, 29].

Similar to the other lncRNAs, appropriate interaction and function of HOATIR depends on the intricate space structures of this molecule. Computational and experimental analyses demonstrated that *HOTAIR* optimally forms a high-order secondary structure, consisting of four independent folded domains. Among these four, two domains have been suggested to interact with transcription factors via particular evolutionary conserved transcription factor binding sites (TFBS): a 200–300 nucleotides length region at the 5′ end of *HOTAIR* (probably containing 11 helices, 8 terminal loops and 3 junctions) and another region with 600–700 nucleotides at the 3′ end of this lincRNA. Presence of these domains in the molecular structure of *HOTAIR* proposes that *HOTAIR* might act as the scaffold, consequently bound to different transcription factors together [30, 31]. The features of this molecule could contribute to *HOTAIR* activity in various processes of cellular development.

### Function of *HOTAIR* in normal development

Generally, an appreciable role is conceived for expression of *HOTAIR* in mammals; although several questions still remain to be elucidated. *HOTAIR* belongs to the conserved genomic region. This region is composed of several coding (including *HOXC11* and *HOXC12*) and non-coding gene members of HOX family, that play essential role in patterning and maintenance of different body compartments, as well as the anterior–posterior axis positional identity [32]. Overall, *HOTAIR* is more conserved in primates than mammalians. This is likely due to some evolutionary procedure. In mammalians, the neighbour genes of *HOTAIR* are highly conserved. Among different mammalian species, *HOTAIR* is composed of two regions including rich conserved (i.e. exons 1, 3–5 as well as domain B of exon 6) and poorly conserved genomic area (exon 2 and exon 6 domain A). HOTAIR transcription nucleotides and structure are highly conserved. Curiously, 5′ domain of the exon 1 and 3′ end of the exon 6 domain B have consistent sequence and structure, binding to multiple transcription factors [18, 19]. These findings suggest that *HOTAIR* might play similar functions among different species. In this regard, despite the limited sequence conservation in some regions, similar role of *HOTAIR* in the regulation of human and mouse *HOXD* genes has been reported [33].

Developmentally, investigations on mouse revealed that *Hotair* is not expressed in the early stage of zygote, when the primary imprinted alleles are methylated [33]. Activity of this lincRNA commences from early stages of embryogenesis, likely soon after four-cells stage, when interaction of coding and non-coding RNA starts to contrive a natural configuration for embryonic development [34]. Thereupon, *Hotair* is expressed in a site specific pattern. Thus, it is transcribed in the genital bud and tail, in addition to the hindlimb bud and posterior trunk within E10.5–E13.5, subsequently contributing to development of lumbosacral region [17, 35]. Moreover, presence of this lincRNA has been observed in some particular mesenchymal cells as well as forelimb and wrist after E11.5 [33]. In human tissues, *HOTAIR* is highly expressed in skin and genital system (including testis, endometrium and prostate respectively). In addition to these tissues, expression of *HOTAIR* has been detected in lymph node, placenta, kidney, fat originating from mesenchymal cells and bladder (data are presented in https://www.ncbi.nlm.nih.gov/gene/100124700#gene-expression, 03 March 2020 according to [36]), however, this expression could be tissue- or cell-dependent in some organs. As a case, among the reproductive system tissues, expression of *HOTAIR* is observed in testis and endometrium, but not ovary. Further investigations have also revealed that *HOTAIR* expression in skin depends on the positional identity of fibroblast. Thus, foreskin and foot fibroblasts could express this lincRNA, in contrast to chest, lung and forearm [17].

Functionally, *HOTAIR* could take part different roles in the cells. These roles are regulated by different molecular mechanisms. Considering the potential capacity of lncRNAs in forming complex (secondary and tertiary) structures, *HOTAIR* could promote or compete (to inhibit function of other molecules), make a scaffold and construct a platform through different RNA-DNA, RNA–RNA (including *HOTAIR*-mRNAs or *HOTAIR*-microRNAs), RNA–protein interactions or epigenetically modification of histones in the cell.

Findings show that *HOTAIR* down-regulates two osteogenic-related genes, *ALPL* and *BMP2*. Additionally, it inactivates several calcification-related genes, proposing the negative role of this non-coding RNA in osteogenesis [37]. By activation of canonical Wnt signalling pathway, β-catenin regulates downstream target genes, contributing to embryonic skeletal development and bone regeneration upon the injury [38]. Curiously, it has been reported that recruiting Wnt/β-catenin signalling pathway could halve the expression of *HOTAIR* [37], further suggesting the likely negative impact of this lincRNA on osteogenesis.

**Fig. 2:**
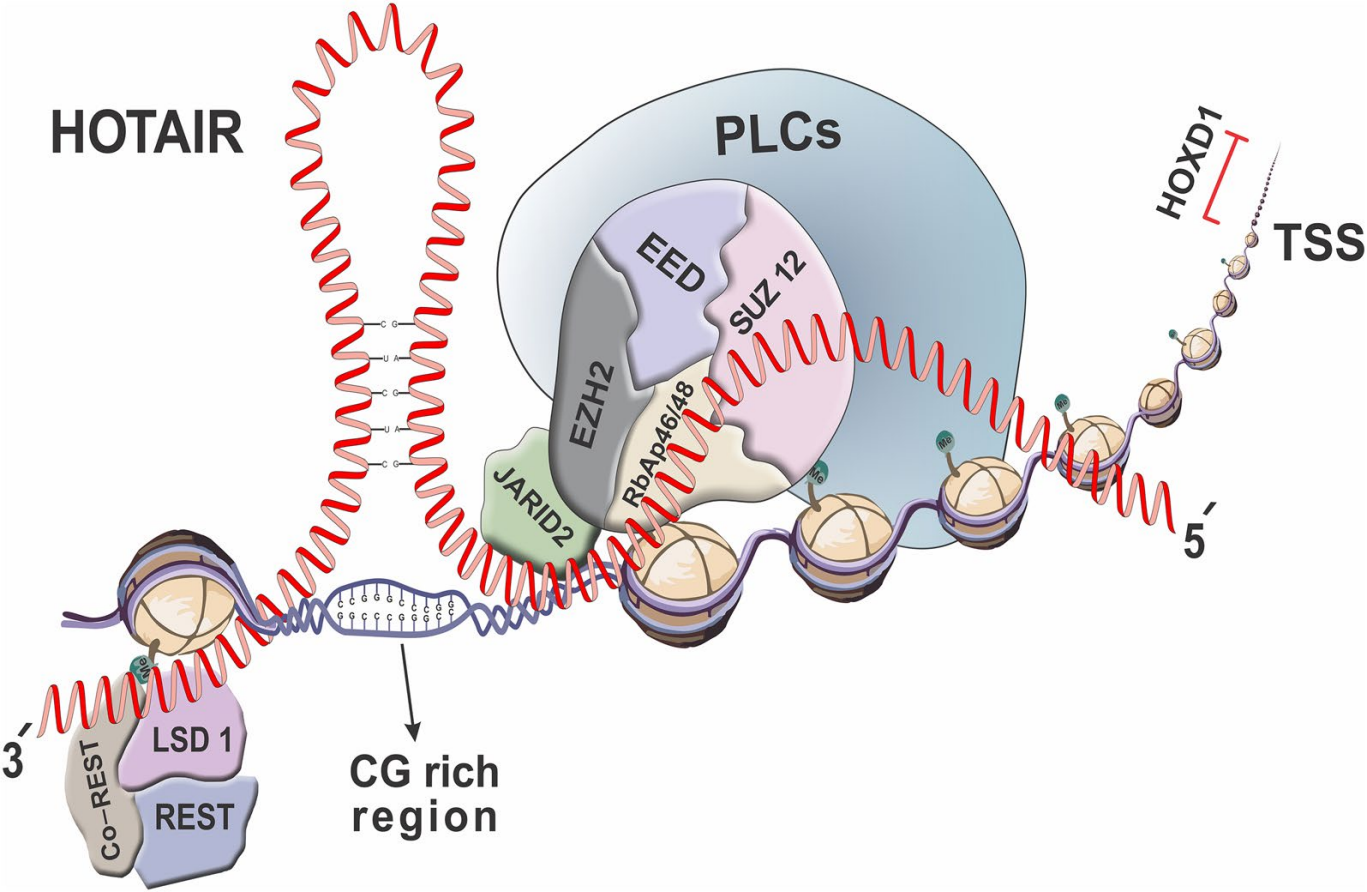
Schematic illustration of *HOTAIR* interactions with PRC2 and LSD1 complexes. To function as scaffold and platform, 5′ end of *HOTAIR* could epigenetically mediates interaction of PRC2 complex (including EZH2, EED, SUZ1 and different RbAp) and other accompaniment proteins with promoter region of the particular genes. Additionally, appropriate function of LSD1/CoREST/REST complex depends on the interaction with 3′ end of *HOTAIR*, leading to de-methylation of H3K4me2 at promoter region and suppression of the corresponding gene expression. TSS; transcription start site

Ability to behave as a molecular scaffold has turned this lincRNA into a crucial component required for regulation of several genes. Amid development, silencing expression of multifarious genes depends on the appropriate function of polycomb repressive complexes (PRCs), including PRC1 and PRC2. As a crucial class of these complexes, PRC2 is composed of four conserved core components (i.e. EZH1/EZH2, SUZ12, EED and histone chaperons, namely RbAp46/RbAp48) as well as several other proteins. To have an optimal activity, AEBP2, JARID2 and polycomb-like family members (PCLs) coordinate in the PRC2 complex (Fig. 2).

AEBP2 is a zinc finger protein, co-localized with PRCs and bind to particular DNA site in some genes [39]. JARID2 is the other constituent of PCR2 complex, binding with EZH2 to enhance activity of the latter complex under defined conditions. This protein is able to bind to DNA with a slight bias towards CG-rich sequences, as a crucial region bound to PRC2 complex [40]. Cooperation of different polycomb-like family members (PCL1, PCL2 and PCL3) with EZH2 (as a histone methyltransferase), sometimes SUZ12 and RbAp46/rbAp48 is required for the PRC2 gene recruitment and regulation of enzyme activity [41]. These combinations could ultimately inhibit expression of many genes by catalysing H3 lysine 27 di- and tri-methylation (H3K27me2 and H3K27me3, respectively) [41]. Nevertheless, PRC2 complex is not individually able to perform the indicated function and this procedure is facilitated by interaction of PRC2 with particular domain of *HOTAIR*. In fact, PRC2 complex could efficiently determine and interact through EZH2/EED or SUZ12 with a fragment at 5′ end of *HOTAIR*, required for recognition of acting site by the other proteins [42–44]. In addition to PRC2, appropriate function of LSD1/CoREST/REST is indebted to the interaction of this protein complex with the *HOTAIR* 3′ domain (nucleotides 1500–2164) [44]. This interaction could lead to de-methylation of H3K4me2 and subsequently promoting repression of the relative genes, including *Hoxd1*, *Hoxd3*, *Hoxd10*, *Hoxd11* and *Hoxd13* [19, 33]. Summarizing the above evidences suggest bi-functional histone modification pattern of *HOTAIR* by methylating/de-methylating particular sites, especially on the benefit of silencing genes.

Apart from the cell nucleus, *HOTAIR* transcript is present in the cytoplasm [45, 46] where it could similarly serve a scaffold role in this compartment by assembling with two E3 ubiquitin ligases (named Dzip3 and Mex3b) through their RNA binding domains. This relatively encourages *HOTAIR* to act as platform facilitating interaction of Dzip3 and Mex3b with Ataxin-1 and Snurportin-1 respectively, causing ubiquitination and rapid decay of them due to recruitment of ubiquitin-mediated proteolysis. This might ultimately lead to cell senescence [47].

Further to the scaffold role, in less than 5% of the cases, *HOTAIR* facilitates interaction of PRC2 and LSD1 complexes by making a platform and bridging these two complexes [42]. It leads to repression of particular genes, through chromatin histone modification of H3K27 and H3K4. As a case, this crucial mechanistic interaction has been determined in epigenetically trans-acting regulation of *HOXD* genes cluster, located about 40 kb far away from *HOTAIR* genomic DNA position. Over the normal development, combination of PRC2 and LSD1 complexes with *Hotair* silences the chromatin state in this region, leading to repression of some HOXD gene members. Loss of *Hotair* could de-repress *HOXD* genes and consequently induce developmental aberration, including homeosis and metacarpal-carpal skeletal malformation in the mouse model [33]. In addition, individual interaction of *HOTAIR* with LSD1 complex, without observing significant impact of PRC2 complex on chromatin modification, could itself repress other specific genes [33]. It has been proposed that *Hotair* might accomplish histone modification through either direct regulation or indirect pleiotropic epigenetic state effects of some imprinted gene loci [33].

**Fig. 3:**
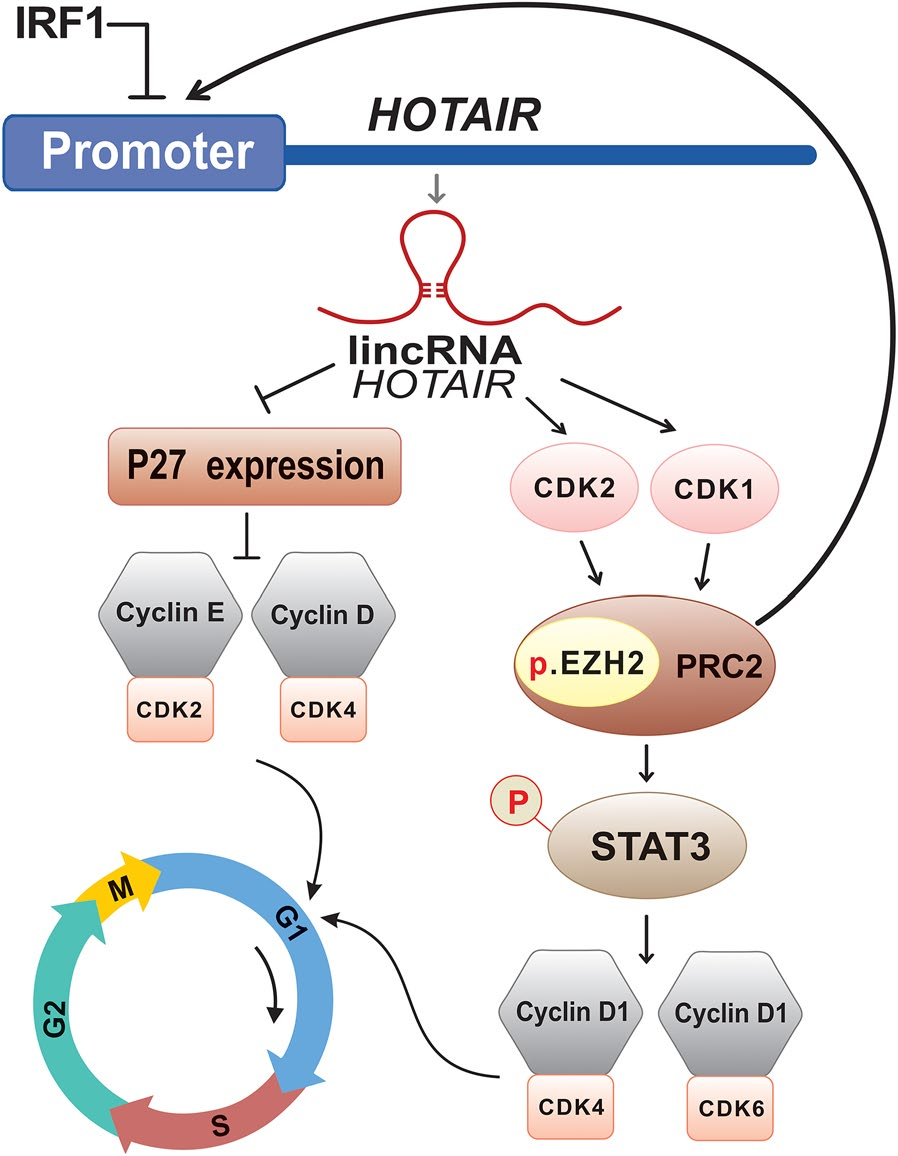
Molecular mechanisms of *HOTAIR* activity to promote cell proliferation. It is proposed that IRF-1 could negatively regulate *HOTAIR* expression. Activity of *HOTAIR* could induce CDK1 and CDK2 activity, leading to phosphorylation of EZH2 in the PRC2. Interaction of *HOTAIR* and PRC2 causes methylation of *STAT3*. This subsequently contributes to STAT3 phosphorylation, as activated form of protein, recruiting Cyclin D1. Additionally, could positively regulate *HOTAIR* activity, by affecting the corresponded gene promoter. Collaboration of Cyclin D1 with CDK4 and CDK6 coordinates in post-translational phosphorylation of some proteins and activity of some necessary transcription factors required for transition of G1 to S cell cycle. Alternatively, activity of *HOTAIR* could down-regulate p27. Defect of this protein could negatively promote activity of Cyclin D-CDK4 and Cyclin E-CDK2. This consequently promotes cell proliferation by contributing to G1 to S phase transition

In addition to making scaffolds and/or platforms to enable interactions of DNA with multiplex proteins, evidences demonstrate the critical effect of *HOTAIR* activity on cell cycle progression and proliferation by regulating different molecules. Transcription of this lincRNA could control expression of different cell cycle-dependent kinase, namely CDK2 and CDK4 as well as Cyclin E and Cyclin D1 [48, 49]. Curiously, it has been shown that *HOTAIR* contributes to the function of Cyclin D1 through activity of STAT3. Although, the mechanism of this procedure still remains unclear, it is proposed that *HOTAIR* coordinates in a molecular pathway leading to promoting proliferation through activation of the CDK1/CDK2/STAT3 signalling cascade (Fig. 3). It has been demonstrated that CDK1 and CDK2 phosphorylate a threonine of EZH2 protein, as an important residue for appropriate function of this protein, in the context of PRC2 complex [50]. Interaction of *HOTAIR* with PRC2 complex promotes methylation in *STAT3*. This methylation plays role in phosphorylation of STAT3 tyrosine residue and activity of this protein [51]. Consequently, Cyclin D1 is recruited by activation of STAT3. Collaboration of Cyclin D1 with CDK4 and CDK6 contributes to post-translational phosphorylation of some proteins and activity of some necessary transcription factors for transition of G1 to S cell cycle [48]. Consistent to this hypothesis, findings revealed that down-regulation of *HOTAIR* could promote G1 cell cycle arrest [49]. So that, loss of this lincRNA promote expression of p27 leading to binding and prohibiting Cyclin D-CDK4 and Cyclin E-CDK2 activities [49] (Fig. 3). In addition to the key effect of *HOTAIR* on the activity of STAT3, findings suggest a positive feedback loop of STAT3 on promoter region of *HOTAIR* and elevating the lincRNA expression [52]. Expression of *HOTAIR* could also be regulated by interaction of IRF-1 transcription factor with promoter of this lincRNA. This mechanism leads to down-regulation of *HOTAIR* [53].

Moreover, *HOTAIR* could function as a competitive endogenous RNA (ceRNA) to regulate several gene expressions through competing with microRNA binding sites, the phenomenon called microRNA sponge. Transcription of *HOTAIR* up-regulates expression of autophagy-related 3 (ATG3) and autophagy-related 7 (ATG7), likely through an indirect ceRNA effect and sponging miRNAs involved in the suppression of these two genes. This associates with promoting activity of autophagy mechanism, consequently leading to protecting cells against proliferation arrest [54].

### *HOTAIR* aberration and breast cancer

Despite the indispensable role of *HOTAIR* in different molecular mechanisms of normal cell development, deregulation of this lincRNA is now determined in several abnormalities like cardiac disease and multiple sclerosis [37, 55]. Up-regulation of this molecule has also been correlated to poor prognosis, invasiveness and metastasis of several tumours, including breast, cervix, endometrial, lung, gastric, hepatocellular and pancreatic cancers as well as glioma. This phenomenon is coordinated by several proteins and noncoding RNA molecules, partially through similar mechanisms in different malignancies (Table 1). Negative impact of hyperactivity of *HOTAIR* has been shown on regulation of *miR*-*141* and *miR*-*326* in glioma cells [56, 57], as well as suppression of *miR*-*141* in breast cancer cells [58]. Enforced transcription of *HOTAIR* could also promote proliferation, invasion and metastasis in gynaecological malignancies and breast cancer [59–61], through different mechanisms including up-regulation of BCL-W and sponging *miR*-*206* [62]. In contrast, activity of *miR*-*330*-*5p* and *miR*-*214*-*3p* could repress *HOTAIR* [63]. GLOBOCAN 2018 reports breast cancer as the second most prevalent and the fourth leading cause of mortality worldwide, due to the malignancies [15]. Curiously, evidences have emphasised the crucial role of *HOTAIR* in cancer cell proliferation and metastasis as well as maintenance of breast cancer stem cells (bCSCs) and EMT. *HOTAIR* is also highly expressed in the CSCs obtained from two breast cancer cell lines: MCF-7 and MB-231, regulating self-renewal, proliferation, colony formation and migration by inhibiting *miR*-*34a* and subsequently up-regulating SOX2 [64]. Considering diverse functions of *HOTAIR*, we briefly discuss some important mechanisms whereby this lincRNA contributes to breast cancer progression.

**Table 1:** Relationship of *HOTAIR* with other non-coding RNAs and proteins

miR ID	Status	Function	Disease	Reference
*miR*-*148a*	Upstream	*miR*-*148* activity represses *HOTAIR* expression by interacting with the corresponding promoter region	Breast cancer	[73]
*miR*-*1*	Upstream	*miR*-*1* supresses expression of *HOTAIR* and MAPK1 activity, prohibiting cell proliferation, invasion and migration	Ovarian cancer	[63]
*miR*-*214*-*3p*	Upstream	*miR*-*214*-*3p* supresses expression of *HOTAIR* and MAPK1 activity	Ovarian cancer	[63]
*miR*-*330*-*5p*	Upstream	*miR*-*330*-*5p* supresses expression of *HOTAIR* and MAPK1 activity	Ovarian cancer	[63]
*miR*-*206*	Competing	Up-regulates BCL-W by sponging *miR*-*206*, elevating cell proliferation rate	Breast cancer	[62]
*miR*-*130a*	Competing	*HOTAIR* represses *miR*-*130a*, likely in a reciprocal negative feedback loop, competition in binding to similar RISC complex	Galbladder cancer	[65]
*miR*-*34a*	Downstream	*HOTAIR* epigenetically supresses *miR*-*34a*, leading to up-regulation of SOX2 and cell proliferation	Breast cancer	[64]
*miR*-*7*	Downstream	*HOTAIR* supresses expression of HOXD10 and subsequent target, *miR*-*7*, ultimately promoting EMT process due to up-regulation of SETDB1 and STAT3	Breast cancer	[78]
*miR*-*20a*-*5p*	Downstream	*HOTAIR* promotes cell growth, mobility and invasiveness via supressing *miR*-*20a*-*5p* and consequently up-regulating *HMGA2*	Breast cancer	[83]
*miR*-*218*	Downstream	*HOTAIR* induces radioresistance by reducing *miR*-*218* expression level and apoptosis	Breast cancer	[102]
*miR*-*138*/*204*/*217*	Downstream	*HOTAIR* directly antagonizes this complex, ultimately overexpressing EZH2 as a target of *miR*-*138*/*217*	Renal cell carcinoma	[84]
*miR*-*200c*	Downstream	*HOTAIR* promotes epigenetic silencing of *miR*-*200c*, Through PRC2-EZH2 complex	Renal cell carcinoma	[84]
*miR*-*141*	Downstream	*HOTAIR* epigenetically inhibits *miR*-*141* expression	Glioma	[56]
*miR*-*326*	Downstream	Inhibit *miR*-*326* activity	Glioma	[57]

#### Oncogenic role of *HOTAIR*

In several malignancies including breast cancer, evidences demonstrated intermediating oncogenic role of *HOTAIR*, on the benefit of c-Myc oncogenic pathway activity. Thus, c-Myc directly interacts with a putative binding site (E-box element) in *HOTAIR* promoter region and positively regulates activity of the latter lincRNA [65]. Subsequently, up-regulation of *HOTAIR* serves a scaffold role in histone demethylase LSD1 activity and directs interaction of HBXIP with c-Myc proteins. This lincRNA/protein complex could consequently mediate transcriptional activity of several c-Myc downstream target genes, including cyclin A, eIF4E and LDHA [66]. Additionally, *HOTAIR* overexpression negatively competes with *miR*-*130a* activity (as a non-coding RNA down-regulated in various malignancies), likely through a reciprocal feedback loop, for binding to the consistent RISC complex [65]. In terms of breast cancer progression, although different investigations have currently demonstrated the individual impact of *HOTAIR*, RISC components (e.g. Argonaute 2) or *miR*-*130a* [67, 68], no report has yet validated any correlation of *HOTAIR* with *miR*-*130a* and RISC, proposing investigation of this objective in future.

#### *HOTAIR* and PRC

It has been shown that hyperactivity of *HOTAIR* could promote breast cancer malignancy through interaction with PRC2 complex [69]. In the same context to embryonic fibroblast, overexpression of *HOTAIR* triggers PRC2 complex in epithelial malignant cells. This leads to H3k27me3 modification of the particular genomic region, deregulation of some genes and subsequently promoting malignant cell invasiveness and metastasis in a PRC2 dependent manner [70].

#### *HOTAIR* and estrogen

Findings obtained from a retrospective clinical study revealed strong association of *HOTAIR* overexpression with risk of metastasis in the estrogen receptor positive (ER^+^) breast cancer patients who diagnosed with primary tumours and received no adjuvant therapy, suggesting this lincRNA as a potential prognostic biomarker in this type of patients [71]. In line with this, studies on the MCF-7 (as an ER^+^/PR^+^ mammary gland epithelial cell line) demonstrated overexpression of *HOTAIR* on the benefits of malignant cell proliferation, growth and invasion. This consequence could be observed due to the estrogen activity, in the form of estradiol (E2). Eestrogen receptor (ER) plays key role in the process of inducing *HOTAIR* activity by E2. Thus, E2 could bind to the estrogen response element (ERE) region of the *HOTAIR* promoter through recruitment of ERs -particularly GPER- and other ER co-regulators, including histone methylases mixed lineage leukemia 1 (MLL1), MLL3 and CREB-binding protein/p300. This mechanism subsequently culminates in hyper-methylation of H3K4me3, histone acetylation, recruitment of RNA polymerase II in *HOTAIR* promoter region and consequently overexpression of this lincRNA [72, 73]. Contrarily, overexpression of *miR*-*148a*, in the absence of ER signalling, down-regulates *HOTAIR* [73]. It has also been shown that *HOTAIR* activity is sufficient to induce ER signalling in the malignant cells with poorly expressed estrogen, likely due to the intermediating action of ER by *HOTAIR* [74].

#### *HOTAIR* and tumour suppressor genes

Overexpression of *HOTAIR* could negatively regulate expression of some tumour suppressor genes, consequently leading to promote breast cancer cell proliferation, invasion and metastasis. It has been shown that down-regulation of *HOTAIR* significantly elevated p53 expression level and reduced expression of AKT and JNK in MCF-7 cell line. Induction of apoptosis, while exhibiting limited metastasis, invasion and proliferation capabilities in this cell line, might likely be due to the cell cycle arrest at G1 phase [64, 75]. Moreover, evidences demonstrated that expression of *HOTAIR* could negatively regulate *p53* and *p21* expressions in MCF-7 and MB-231 bCSCs, leading to cell cycle entry and proliferation, while down-regulation of this lincRNA caused activation of *p21* and cell cycle arrest at G1 phase, likely by inhibiting CDK1, CDK2, CDK4 and CDK6 [64].

Demonstrating the negative role of BRCA1 in PRC2 complex activity [76] raised the question whether this crucial tumour suppressor gene could have any potential correlation with *HOTAIR*? Investigations showed that *HOTAIR* could carry action against BRCA1 to positively regulate PRC2 complex in breast cancer. In this mechanism, binding of BRCA1 to EZH2 -among the PRC2 complex- prohibits interaction of the latter protein with *HOTAIR* in malignant cells. With loss of BRCA1, *HOTAIR* competitively interacts with EZH2 via similar binding site to BRCA1, culminating in hypermethylation of H3K27me3 and PRC2 occupancy of the corresponding target sites in the breast luminal epithelial cancer cells [77].

Further studies also indicated negative effect of *HOTAIR* on *miR*-*7* activity, in MCF-7 and MB-231 bCSCs cell lines. As a tumour suppressor microRNA, activity of *miR*-*7* could inhibit oncogenic behaviour of SET domain bifurcated histone lysine methyltransferase I (SETDB1) in breast cancer. Negative regulation of *miR*-*7,* through *HOTAIR* activity, contributes to malignant cell proliferation, invasion and metastasis [78]. In addition to the indicated tumour suppressor genes, it has been determined that *HOTAIR* can mediate interaction of EZH2 with the specific region of *PTEN* [79]. Thus, this complex regulates promoter methylation of *PTEN* and repression of the gene expression, ultimately leading to induction of PI3K signalling pathway [80].

#### *HOTAIR* and oncogenes

Expression of *HOTAIR* in invasive malignant cells, on one hand, and down-regulation of this lincRNA in the malignant cells which have undergone apoptosis, on the other hand, propose *HOTAIR* direct/indirect role in positively modulating property of multiple oncogenes. HER2 is one of the crucial oncogenic biomarkers in the particular subgroup of breast tumours. Investigations demonstrated that *HOTAIR* is significantly up-regulated in HER2^+^ breast cancer cells [81]. No study has yet been performed to validate the correlation of *HOTAIR* and *HER2* in breast cancer. However, positive effect of this lincRNA has been determined on the regulation of HER2 in gastric carcinoma cells. It was shown that *HOTAIR* acts as a ceRNA to sponge *miR*-*331*-*3p* and *miR*-*124*. Interestingly, *HER2* expression is directly targeted by *miR*-*331*-*3p*. Thus, a positive correlation was determined between *HOTAIR* and *HER2* expression in the HER2^+^ gastric malignancies [82], suggesting consistent mode of interaction in HER2^+^ breast cancer cells.

High-mobility group AT-hook 2 (HMGA2) is the other oncogenic protein highly expressed in breast malignancies. A positive regulation of *HMGA2* was observed by activity of *HOTAIR*, as ceRNA for *miR*-*20a*-*5p* in breast cancer cells. This leads to overexpression of HMGA2, binding to AT-rich regions in DNA and chromatin modification to facilitate some transcriptional enhancer actions [83].

Activity of estrogen receptor beta (ERβ), as a key factor in progression and invasion of many cancer types, has been reported to up-regulate *HOTAIR* in renal cell carcinoma. Antagonizing behaviour of this lincRNA sponge the activity of *miR*-*138*/*204*/*217* complex, among which *miR*-*138*/*217* negatively regulate EZH2. This leads to up-regulation of EZH2 and epigenetically promoter silencing of *miR*-*200c* downstream of *HOTAIR*, consequently directing malignant cells to proliferation and invasion [84]. Interestingly, ERβ could play crucial role in breast cancer cells progression, particularly EMT and metastasis [85]. This suggests potential correlation of *HOTAIR* and ERβ in breast cancer and subsequently downstream molecules, *miR*-*138*/*204*/*217* and *miR*-*200c*, in breast cancer, although, it still remains to be investigated.

#### Epithelial mesenchymal transition and *HOTAIR*

**Fig. 4:**
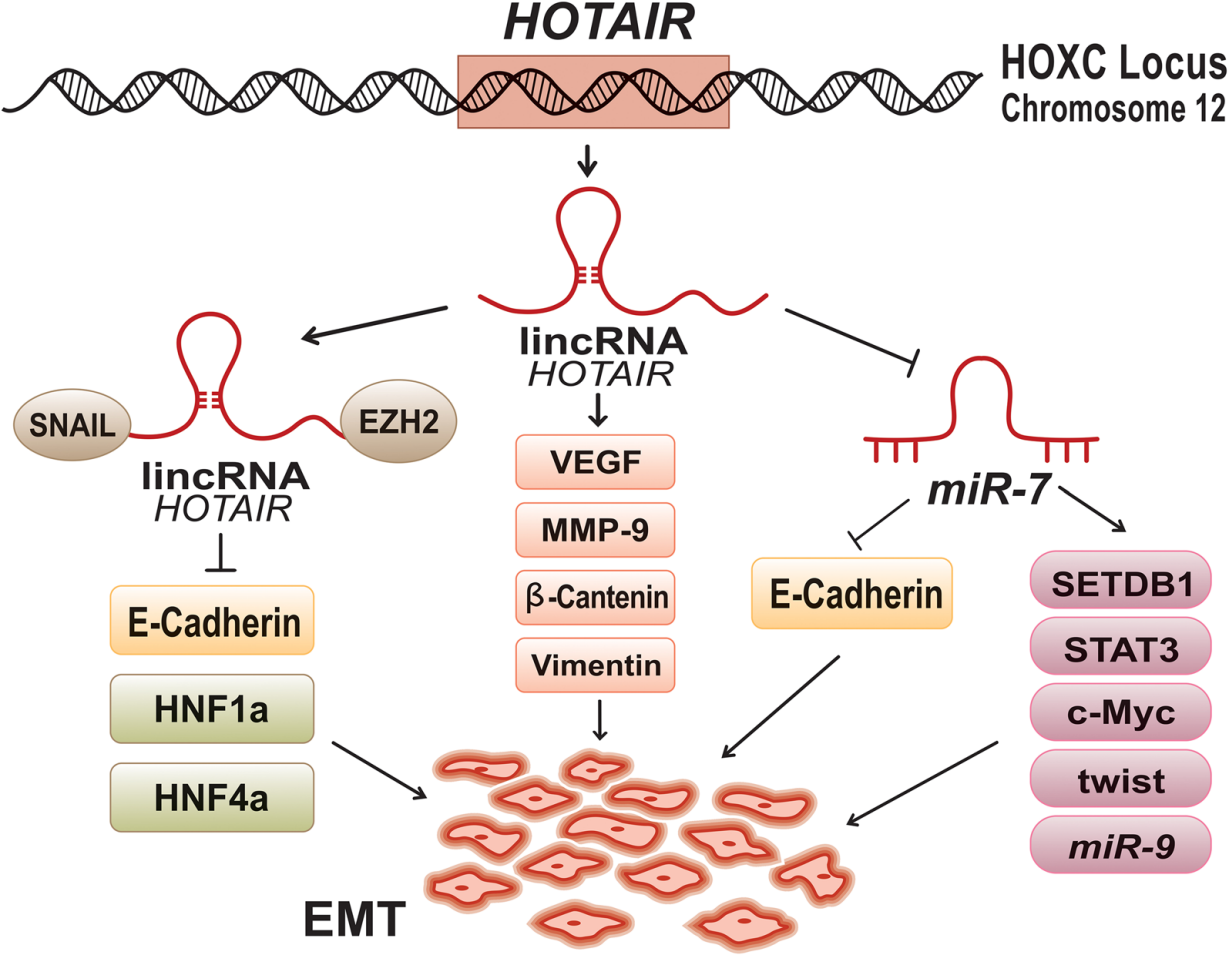
Effect of *HOTAIR* on epithelial-mesenchymal transition. *HOTAIR* could promote epithelial-mesenchymal transition (EMT) through at least three pathways. lincRNA *HOTAIR* activity could indirectly inhibit *miR*-*7*. This leads to overexpression of SETDB1, STAT3, c-Myc, twist and *miR*-*9*, while E-cadherin is down-regulated. *HOTAIR* activity could also promote prometastatic activity of cancer cells by regulating VEGF, MMP-9, β-catenin and Vimentin. Moreover, lincRNA *HOTAIR* could coordinate in tripartite SNAIl/*HOTAIR*/EZH2 complex. This complex involves in general chromatin modification to inhibit expression of the genes involved in epithelial formation (e.g. *HNF4α*, *HNF1α* and *E*-*cadherin*)

As an essential stage, EMT is involved in tumour invasion and metastasis. The important role of *HOTAIR* in metastasis raises the question whether there is any potential correlation between this lincRNA and EMT in breast cancer patients? Investigations showed that *HOTAIR* could indirectly inhibit *miR*-*7* in bCSCs obtained from MCF-7 and MB-231 cell lines. This leads to overexpression of SETDB1, STAT3, c-Myc, twist and *miR*-*9* [78] and down-regulation of E-cadherin [78, 86] on the benefit of EMT process (Fig. 4). *HOTAIR* also contributes to EMT and prometastatic activity of malignant cell via regulation of VEGF, MMP-9, β-cantenin and Vimentin [87]. Additionally, *HOTAIR* up-regulates expression of SNAIL, as a master regulator of EMT pathway, in breast cancer [70]. Subsequently, this lincRNA could mediate establishment of tripartite SNAIL/*HOTAIR*/EZH2 complex. Function of this constructive complex conveys a general chromatin modification to repress epithelial genes (like *HNF4a*, *HNF1a* and *E*-*cadherin*) in the EMT frame [88] (Fig. 4).

Negative correlation of *HOTAIR* and *miR*-*148a* has also been demonstrated to cause EMT and metastasis. In the triple negative and ER^+^ breast cancer cells, E2-GPER signal promotes *HOTAIR* expression. A particular site has been discerned to facilitate binding of this lincRNA to *miR*-*148a*. This leads to *miR*-*148a* sponge by *HOTAIR* [73]. Inhibition of this microRNA negatively regulates SNAIL2 expression. This subsequently might lead to down-regulation of E-cadherin, Caludin-1 and several adhesion molecules, consequently promoting EMT event and metastasis in malignancies [89].

Previous study on hepatocellular malignancy highlighted the remarkable effect of *HOTAIR* on the blockage of RNA binding motif protein 38 (RBM38), as a tumour suppressor gene [90]. Adding to this, disrupted expression level of RBM38 in breast cancer due to the silencing E-box element promoter region by SNAIL [91], propose the indirect effect of *HOTAIR* on regulation of this tumour suppressor gene. Inhibition of RBM38 activity could destabilize zonula occludens-1, consequently leading to induction of EMT and metastasis [91].

Curiously, evidences emphasize the crucial role of *HOTAIR* overexpression in breast cancer radioresistance through EMT induction. Using two different experiments, it has been demonstrated that *HOTAIR* activity reduced radiosensitivity of MB231 and SKBR3 breast cancer cell lines by down-regulation of the HOXD10 tumour suppressor gene and the corresponding pathway, PI3K/AKT-Bad [92, 93]. Down-regulation of HOXD10 also inhibits expression of *miR*-*7* and subsequently a histone methyltransferase, SETDB1, inducing STAT3 function in EMT frame [78].

Considering the critical role of *HOTAIR* in promoting breast cancer development, through different mechanisms of action, we further discuss the potential relationship of *HOTAIR* with response to different combinational therapeutic agents, in the next section.

### *HOTAIR* and treatment approaches

To date, resistance to different therapeutic approaches is one of the most important challenges of breast cancer treatment. Several evidences emphasize the crucial role of *HOTAIR* in breast cancer resistance [74, 92, 94]. Additionally, administrating some agents could down-regulate *HOTAIR* activity. Regarding that some therapeutic agents are commonly used in diverse malignancies, here, we generally discuss the relationship of *HOTAIR* to different treatment approaches in the cancer resistant and sensitive cells (Table 2).

**Table 2:** Effect of some chemical drugs on the expression of *HOTAIR*

Component	Drug category	Effect on *HOTAIR*	Comment	References
Calycosin	Phytostrogen isoflavon	Down-regulation	Induces apoptosis by down-regulating phosphorylation of *HOTAIR* upstream target, Akt	[116]
Genistein	Soy isofalvone	Down-regulation	Represses *HOTAIR* as well as NF-κB and Akt signalling pathways, while it overexpresses *miR*-*141*	[116, 119]
BIO	Genistein nano-suspension	Down-regulation	Inhibits GSK3β and induces β-Catenin signalling, leading to down-regulation of *HOTAIR*	[37]
Delphinidin-3-glucoside	Anthocyanidin	Down-regulation	Induces apoptosis via activation of IRF1 and repression of Akt	[53]
Imatinib + Lapatinib	Anti-neoplastic agent	Down-regulation	Synergistically supress β-Catenin and subsequently *HOTAIR* expression	[117]
BML-284	Wnt agonist	Down-regulation	Induces Wnt/β-Catenin signalling pathway and repression of *HOTAIR*	[37]
Bisphenol-A	estrogenic endocrine disrupting chemical	Up-regulation	Interferes with normal estrogen signalling pathway, leading to expression of *HOTAIR* by inducing the corresponding ERE promoter, in addition to particular histone modifications	[95]
Diethylstilbestrol	Synthetic estrogen	Up-regulation	Involved in normal estrogen signalling pathway and *HOTAIR* expression, through interaction with the corresponding ERE promoter and particular histone modifications	[95]
Gemcitabine	Anti-metabolite agents	Up-regulation	Through unknown mechanism, this agent up-regulates *HOTAIR* causing further malignant cell proliferation, self-renewal and migration	[104]

#### *HOTAIR* and hormone therapy

Accumulating data indicate the potential role of *HOTAIR* in prohibiting the effect of several hormonal therapeutic agents. As previously indicated, *HOTAIR* activity directly fosters ER signalling in ER^+^ breast cancer cells to develop invasiveness and metastasis. Mechanistically, activity of *HOTAIR* elevates ER occupancy on chromatin and regulates the corresponding downstream genes. This mechanism further encourages drug-resistance in the cancer patients treated with Tamoxifen (as an ER competitive antagonist). It is proposed that *HOTAIR* could promote ER activity in the Tamoxifen resistant malignant breast cancer cells with lack of estrogen [74]. Additionally, administration of Bisphenol-A (BPA), as an endocrine disrupting chemical, and Diethylstilbestrol (DES), as a synthetic estrogen, facilitates *HOTAIR* activation both in vitro and in vivo by modifying histone methylation/acetylation status at the corresponding promoter, particularly ERE, region. This process is mediated by binding ERs, MLL1 and MLL3 to the *HOTAIR* promoter EREs, chromatin modification and consequently *HOTAIR* activity [95]. Further investigations on prostate cancer indicated castration-resistance due to the overexpression of *HOTAIR*. Thus, activity of this lincRNA induces a distinct mode of androgen receptor (*AR*) gene regulation through interaction with MDM2 (an E3 ubiquitin ligase), prohibiting the respected protein ubiquitination and consequently AR degradation; while, overexpression of *HOTAIR* is sufficient to activate androgen-independent AR and promote drug-resistance in the absence of androgen, through AR-mediated transcriptional pathway [96].

#### *HOTAIR* and radiotherapy

As an essential method of adjuvant therapy, radiation has been linked to *HOTAIR* in different cancers. Activity of this lincRNA could minimize radiosensitivity of colorectal cancer. Down-regulation of *HOTAIR*, in addition to treating colorectal malignant cells with irradiation, reduces MMP-2 and MMP-9 expressions, as two important factors involved in EMT and metastasis [97]. Investigations on pancreatic ductal adenocarcinoma cells revealed expression of *HOTAIR*. Expression of this lincRNA enhances radioresistance via negatively regulation of Wnt inhibitory factor 1 (WIF-1), culminating in further proliferation rate and less apoptosis [98]. Overexpression of *HOTAIR* can also increase HIF-1α expression in cervical cancer. Thus, HIF-1α induces malignant cell resistance to the radiation [99] (Table 3). Additionally, up-regulation of *HOTAIR* has been suggested to induce radioresistance in HeLa and C33A cells, in a competition, by prohibiting *p21* activity, while up-regulation of *p21* could neutralize the negative effect of *HOTAIR* activity on cell resistance against ionizing radiation [100].

**Table 3:** Effect of HOTAIR overexpression on radiosensitivity of different cancer types

	Type of malignancy	Radiosensitivity	Molecular mechanism	Mode of action	Reference
HOTAIR overexpression	Colorectal cancer	Reduction	MMP-2 and MMP-9 increase	Promoting EMT and metastasis	[97]
	Pancreatic ductal carcinoma	Reduction	(WIF-1) decrease	Promoting proliferation, inhibiting apoptosis	[98]
	Cervical cancer	Reduction	HIF-1α increase, P21 decrease	Induce hypoxia and radioresistance	[99, 100]

In breast cancer cells, studies demonstrated relation of *HOTAIR* expression level with metastasis free survival and enhancing rim fraction (ERF) radiogenomics score [91, 101]. It can also contribute to radioresistance by function as ceRNA. Mechanistically, *HOTAIR* expression competitively inhibits *miR*-*218* activity. Down-regulation of *HOTAIR* leads to radiosensitivity of breast cancer cells, induction of DNA damage, cell cycle arrest and apoptosis by recruiting *miR*-*218* [102].

#### *HOTAIR* and chemotherapy

Similar to radiotherapy and hormone therapy, *HOTAIR* activity could deregulate the mechanism of several commonly used chemotherapeutic such as carboplatin and gemcitabine in breast and other types of cancer [103–105]. Investigations on the stage II/III breast cancer patients who undergone neoadjuvant treatment, using taxan-based and/or anthracyclin-based chemical agents, showed correlation of the drug response to the level of circulating *HOTAIR*. Thus, more drug-resistance was observed in the patients with higher level of *HOTAIR* and conversely less chemo-resistance effect was determined in the patients with lower *HOTAIR* expression level [106].

Additionally, *HOTAIR* associates with Cisplatin drug resistance in gastric cancer via blocking expression of *miR*-*126* and recruiting VEGFA/PI3K/AKT/MRP1 or PIK3R2/PI3K/AKT/MRP1 pathway. This process induces G1/S phase cell cycle progression and cell proliferation, but restrains malignant cell apoptosis [107]. *HOTAIR* also plays role in Cisplatin chemoresistance of lung adenocarcinoma cells by interacting with EZH2 and suppressing *p21*. This consequently prohibits cell cycle arrest at G0/G1 phase and apoptosis, while induces cell proliferation [108]. *HOTAIR* is also positively involved in chemoresistance of the small cell lung cancer cells treated with Cisplatin, Adriamycin and Etoposide, through epigenetically suppressing *HOXA1* expression. Mechanistically, it has been proposed that *HOTAIR* could up-regulate activity of two DNA methyltrasnferases, DNMT1 and DNMT3b, combination of which hypermethylates *HOXA1* gene promoter CpG islands and consequently silences the corresponding gene expression. Down-regulation of *HOTAIR* improves sensitivity of these malignant cells to the indicated chemical agents, through up-regulating *HOXA1* expression, leading to tumour growth contraction as well as induction of cell cycle arrest and apoptosis [109]. In line with the presented malignancies, evidences revealed the effect of *HOTAIR* expression on promoting Cisplatin resistance in ovarian cancer [110]. This process is mediated by activating Wnt/β-Catenin signalling pathway, promoting proliferation and cell cycle progression, while it is arrested by Cisplatin at G1 phase with defect of *HOTAIR* [111]. Recently, investigations on colorectal cancer revealed that *HOTAIR* targets Wnt/β-Catenin pathway by sponging *miR*-*203*-*3p*, while presence of this microRNA can cause cell sensitivity to Cisplatin and Paclitaxel by blocking Wnt/β-Catenin pathway [112].

Moreover, it has been shown that *HOTAIR* is highly expressed in the 5FU drug resistant colorectal cancer cells. Thus, *HOTAIR* recruits EZH2 protein and this complex supress *miR*-*218*-*2* by interacting with the corresponding promoter region. Lack of *miR*-*281*-*2* could consequently lead to activation of NF-κB/TS signalling pathway [113]. Similarly, evidences demonstrate that overexpression of *HOTAIR* could cause platinum resistance of ovarian cancer by inducing NF-κB and downstream target gene, interleukin-6 (*IL*-*6*). Activity of the latter protein promotes BCL2, BCL-XL and XIAP to inhibit apoptosis 61). Consistently, activity of IL-6 has been linked to the resistance of Cisplatin and Carboplatin drugs in ovarian cancer cells [114]. Subsequently, another evidences further validated the correlation of *HOTAIR* expression with Carboplatin resistance in ovarian cancer [115].

These data emphasize the crucial role of *HOTAIR* in promoting resistance against some therapeutic agents, as an oncogenic lincRNA. In contrast to the above subjects, some chemical agents have thus far been recognized to induce sensitivity and cytotoxicity in the malignant cells, through negative regulation of *HOTAIR* (Table 2). In this context, evidences demonstrated that administration of isoflavone-based agents, including Calycosin and Genistein, play dose-dependently anti-tumour roles in breast cancer cells by inhibiting *HOTAIR* expression and phosphorylation of Akt causing suppression of PI3K/AkT signalling pathway and consequently defect of apoptosis inhibitors, BCL-2 family and casepases. This mechanism inhibits malignant cell proliferation and induces apoptosis [116, 117]. Combination of Imatinib and Lapatinib compounds could also been reported to repress *HOTAIR* expression in triple negative BC cells [118].

Recently, Newphew and colleagues has also been able to restore the chemical effect of platinum in the chemoresistant breast and ovarian cancer cells using a polypeptide nucleic acids (PNAs)-based approach, blocking EZH2 domain of *HOTAIR*. In this experiment, the PNAs inhibited *HOTAIR*-EZH2 activity, subsequently reducing expression of NF-κB and corresponding proteins, IL-6 and MMP-9, which consequently culminated in decrease of tumour formation and improvement of survival chance [119]. Therefore, these findings suggest *HOTAIR*, as a potential therapeutic target to prohibit tumourigenesis progress.

## Conclusions and future prospective

Thanks to the instrumental developments and technology advances, some integral missions of lncRNAs in physiological and developmental systems of human cells have hitherto been discovered, although perturbation of these molecules can lead to different abnormalities, including cell malignancies. The present review posits that *HOTAIR* plays a significant role in normal development and survival of diverse tissue cells. Nonetheless, inappropriate expression of this lincRNA is able to promote malignant cell progression by deregulation of several crucial pathways. Here, we highlighted the impact of unfitting expression of *HOTAIR* in the survival and progression of breast cancer cells; in some cases, we also implicated the role of HOTAIR in other types of cancer to elucidate potential role of this lincRNA in breast cancer. It was implicated that *HOTAIR* could mimic oncogenic behaviour in several breast cancer patients, leading to the aberration of several molecular pathways, towards the malignant cell proliferation, invasion, EMT, metastasis as well as resistance against different therapeutic agents. Thus, finding an efficient therapeutic strategy to direct malignant cell apoptosis through down-regulation of *HOTAIR* could be considered as a future plan. In other words, understanding the underlying function and mechanisms of this lincRNA might not only suggest *HOTAIR* as a potential biomarker in prediction of breast cancer susceptibility and prognosis of the disease, but also help clinicians more appropriately perform patient management and find the most beneficial therapeutic approaches in the frame of personalized medicine.

## Data Availability

Data sharing is not applicable to this article, as no datasets were generated or analysed during the current study.

## Abbreviations

AR: Androgen receptor
ATG3: Autophagy-related 3
ATG7: Autophagy-related 7
bCSC: Breast cancer stem cell
BPA: Bisphenol-A
ceRNA: Competitive endogenous RNA
DES: Diethylstilbestrol
E2: Estradiol
EMT: Epithelial–mesenchymal transition
ER: Estrogen receptor
ERE: Estrogen response element
ERE: Estrogen response element
ERF: Enhancing rim fraction
ERβ: Estrogen receptor beta
H3K27me2: H3 lysine 27 di-methylation
H3K27me3: H3 lysine 27 tri-methylation
HMGA2: High-mobility group AT-hook 2
HOTAIR: *HOX* transcript antisense intergenic RNA
IL-6: Interleukin-6
IRF1: Interferon regulatory factor 1
lincRNA: Long intergenic non-coding RNA
lncRNA: Long non-coding RNA
MLL1: Mixed lineage leukemia 1
ncRNA: Non-coding RNA
PCL: Polycomb-like
PNAs: Polypeptide nucleic acids
PRC: Polycomb repressive complex
qRT-PCR: Quantitative reverse-transcription PCR
RBM38: RNA binding motif protein 38
SETDB1: SET domain bifurcated histone lysine methyltransferase I
SNP: Single nucleotide polymorphism
TFBS: Transcription factor binding sites
TSS: Transcription start site
WIF-1: Wnt inhibitory factor 1

## References

[1] Hajjari M, Salavaty A (2015). HOTAIR: an oncogenic long non-coding RNA in different cancers. Cancer Biol Med..

[2] Santosh B, Varshney A, Yadava PK (2015). Non-coding RNAs: biological functions and applications. Cell Biochem Funct.

[3] Bhan A, Soleimani M, Mandal SS (2017). Long noncoding RNA and cancer: a new paradigm. Cancer Res.

[4] O’Leary VB, Hain S, Maugg D, Smida J, Azimzadeh O, Tapio S (2017). Long non-coding RNA PARTICLE bridges histone and DNA methylation. Sci Rep.

[5] Song Y, Wang R, Li L-W, Liu X, Wang Y-F, Wang Q-X (2019). Long non-coding RNA HOTAIR mediates the switching of histone H3 lysine 27 acetylation to methylation to promote epithelial-to-mesenchymal transition in gastric cancer. Int J Oncol.

[6] Durut N, Scheid OM (2019). The role of noncoding RNAs in double-strand break repair. Front Plant Sci.

[7] Koch L (2017). Non-coding RNA: a protective role for TERRA at telomeres. Nat Rev Genet.

[8] Fernandes JC, Acuña SM, Aoki JI, Floeter-Winter LM, Muxel SM (2019). Long non-coding RNAs in the regulation of gene expression: physiology and disease. Non-coding RNA..

[9] Lu W, Yu J, Shi F, Zhang J, Huang R, Yin S (2019). The long non-coding RNA Snhg3 is essential for mouse embryonic stem cell self-renewal and pluripotency. Stem Cell Res Ther.

[10] Yan Z, Ruoyu L, Xing L, Hua L, Yi Z, Yaqin P (2020). Long non-coding RNA GAS5 regulates the growth and metastasis of human cervical cancer cells via induction of apoptosis and cell cycle arrest. Archiv Biochem Biophys.

[11] Zhao H-Y, Zhang S-T, Cheng X, Li H-M, Zhang L, He H (2019). Long non-coding RNA GAS5 promotes PC12 cells differentiation into Tuj1-positive neuron-like cells and induces cell cycle arrest. Neural Regen Res.

[12] Bhan A, Mandal SS (2014). Long noncoding RNAs: emerging stars in gene regulation, epigenetics and human disease. ChemMedChem.

[13] Al-Rugeebah A, Alanazi M, Parine NR (2019). MEG3: an oncogenic long non-coding RNA in different cancers. Pathol Oncol Res.

[14] Liu Y, Sharma S, Watabe K (2015). Roles of lncRNA in breast cancer. Front Biosci (Schol Ed)..

[15] Bray F, Ferlay J, Soerjomataram I, Siegel RL, Torre LA, Jemal A (2018). Global cancer statistics 2018: GLOBOCAN estimates of incidence and mortality worldwide for 36 cancers in 185 countries. CA Cancer J Clin.

[16] Zhou S, He Y, Yang S, Hu J, Zhang Q, Chen W (2018). The regulatory roles of lncRNAs in the process of breast cancer invasion and metastasis. Biosci Rep..

[17] Rinn JL, Kertesz M, Wang JK, Squazzo SL, Xu X, Brugmann SA (2007). Functional demarcation of active and silent chromatin domains in human HOX loci by noncoding RNAs. Cell.

[18] He S, Liu S, Zhu H (2011). The sequence, structure and evolutionary features of HOTAIR in mammals. BMC Evol Biol.

[19] Cai B, Song XQ, Cai JP, Zhang S (2014). HOTAIR: a cancer-related long non-coding RNA. Neoplasma..

[20] Loewen G, Jayawickramarajah J, Zhuo Y, Shan B (2014). Functions of lncRNA HOTAIR in lung cancer. J Hematol Oncol.

[21] Hajjari M, Rahnama S (2017). HOTAIR long non-coding RNA: characterizing the locus features by the in silico approaches. Genom Inf.

[22] Pawlowska E, Szczepanska J, Blasiak J (2017). The long noncoding RNA HOTAIR in breast cancer: does autophagy play a role?. Int J Mol Sci.

[23] Yang G, Zhang S, Gao F, Liu Z, Lu M, Peng S (2014). Osteopontin enhances the expression of HOTAIR in cancer cells via IRF1. Biochem Biophys Acta.

[24] Lu L, Zhu G, Zhang C, Deng Q, Katsaros D, Mayne ST (2012). Association of large noncoding RNA HOTAIR expression and its downstream intergenic CpG island methylation with survival in breast cancer. Breast Cancer Res Treat.

[25] Hassanzarei S, Hashemi M, Sattarifard H, Hashemi SM, Bahari G, Ghavami S (2017). Genetic polymorphisms of HOTAIR gene are associated with the risk of breast cancer in a sample of southeast Iranian population. Tumour Biol.

[26] Zhang X, Zhou L, Fu G, Sun F, Shi J, Wei J (2014). The identification of an ESCC susceptibility SNP rs920778 that regulates the expression of lncRNA HOTAIR via a novel intronic enhancer. Carcinogenesis.

[27] Qiu H, Liu Q, Li J, Wang X, Wang Y, Yuan Z (2016). Analysis of the association of HOTAIR single nucleotide polymorphism (rs920778) and risk of cervical cancer. APMIS.

[28] Bayram S, Sumbul AT, Batmaci CY, Genc A (2015). Effect of HOTAIR rs920778 polymorphism on breast cancer susceptibility and clinicopathologic features in a Turkish population. Tumour Biol.

[29] Bayram S, Sumbul AT, Dadas E (2016). A functional HOTAIR rs12826786 C>T polymorphism is associated with breast cancer susceptibility and poor clinicopathological characteristics in a Turkish population: a hospital-based case-control study. Tumour Biol.

[30] Wang H, Zheng H, Wang C, Lu X, Zhao X, Li X (2017). Insight into HOTAIR structural features and functions as landing pads for transcription regulation proteins. Biochem Biophys Res Commun.

[31] Somarowthu S, Legiewicz M, Chillon I, Marcia M, Liu F, Pyle AM (2015). HOTAIR forms an intricate and modular secondary structure. Mol Cell.

[32] Selleri L, Bartolomei MS, Bickmore WA, He L, Stubbs L, Reik W (2016). A Hox-embedded long noncoding RNA: is it all hot air?. PLoS Genet.

[33] Li L, Liu B, Wapinski OL, Tsai MC, Qu K, Zhang J (2013). Targeted disruption of Hotair leads to homeotic transformation and gene derepression. Cell Rep.

[34] Hammer T, Lohbauer S (2010). The master of genomic programs: HOTAIR in cellular development and cancer metastasis. Aquila..

[35] Schorderet P, Duboule D (2011). Structural and functional differences in the long non-coding RNA hotair in mouse and human. PLoS Genet.

[36] Fagerberg L, Hallstrom BM, Oksvold P, Kampf C, Djureinovic D, Odeberg J (2014). Analysis of the human tissue-specific expression by genome-wide integration of transcriptomics and antibody-based proteomics. MCP.

[37] Carrion K, Dyo J, Patel V, Sasik R, Mohamed SA, Hardiman G (2014). The long non-coding HOTAIR is modulated by cyclic stretch and WNT/beta-CATENIN in human aortic valve cells and is a novel repressor of calcification genes. PLoS ONE.

[38] Long H, Sun B, Cheng L, Zhao S, Zhu Y, Zhao R (2017). miR-139-5p represses BMSC osteogenesis via targeting Wnt/beta-catenin signaling pathway. DNA Cell Biol.

[39] Kim H, Kang K, Kim J (2009). AEBP2 as a potential targeting protein for polycomb repression complex PRC2. Nucleic Acids Res.

[40] Portoso M, Ragazzini R, Brencic Z, Moiani A, Michaud A, Vassilev I (2017). PRC2 is dispensable for HOTAIR-mediated transcriptional repression. EMBO J.

[41] Margueron R, Reinberg D (2011). The Polycomb complex PRC2 and its mark in life. Nature.

[42] Wu Y, Zhang L, Wang Y, Li H, Ren X, Wei F (2014). Long noncoding RNA HOTAIR involvement in cancer. Tumour Biol.

[43] Wu L, Murat P, Matak-Vinkovic D, Murrell A, Balasubramanian S (2013). Binding interactions between long noncoding RNA HOTAIR and PRC2 proteins. Biochemistry.

[44] Tsai MC, Manor O, Wan Y, Mosammaparast N, Wang JK, Lan F (2010). Long noncoding RNA as modular scaffold of histone modification complexes. Science.

[45] Dodd DW, Gagnon KT, Corey DR (2013). Digital quantitation of potential therapeutic target RNAs. Nucleic Acid Ther..

[46] Cantile M, Scognamiglio G, Marra L, Aquino G, Botti C, Falcone MR (2017). HOTAIR role in melanoma progression and its identification in the blood of patients with advanced disease. J Cell Physiol.

[47] Yoon JH, Abdelmohsen K, Kim J, Yang X, Martindale JL, Tominaga-Yamanaka K (2013). Scaffold function of long non-coding RNA HOTAIR in protein ubiquitination. Nat Commun..

[48] Zhou JJ, Cheng D, He XY, Meng Z, Li WZ, Chen RF (2017). Knockdown of Hotair suppresses proliferation and cell cycle progression in hepatocellular carcinoma cell by downregulating CCND1 expression. Mol Med Rep.

[49] Li E, Zhao Z, Ma B, Zhang J (2017). Long noncoding RNA HOTAIR promotes the proliferation and metastasis of osteosarcoma cells through the AKT/mTOR signaling pathway. Exp Ther Med..

[50] Zeng X, Chen S, Huang H (2011). Phosphorylation of EZH2 by CDK1 and CDK2: a possible regulatory mechanism of transmission of the H3K27me3 epigenetic mark through cell divisions. Cell Cycle.

[51] Kim E, Kim M, Woo DH, Shin Y, Shin J, Chang N (2013). Phosphorylation of EZH2 activates STAT3 signaling via STAT3 methylation and promotes tumorigenicity of glioblastoma stem-like cells. Cancer Cell.

[52] Liu Y, Luo F, Xu Y, Wang B, Zhao Y, Xu W (2015). Epithelial-mesenchymal transition and cancer stem cells, mediated by a long non-coding RNA, HOTAIR, are involved in cell malignant transformation induced by cigarette smoke extract. Toxicol Appl Pharmacol.

[53] Yang X, Luo E, Liu X, Han B, Yu X, Peng X (2016). Delphinidin-3-glucoside suppresses breast carcinogenesis by inactivating the Akt/HOTAIR signaling pathway. BMC Cancer..

[54] Yang L, Zhang X, Li H, Liu J (2016). The long noncoding RNA HOTAIR activates autophagy by upregulating ATG3 and ATG7 in hepatocellular carcinoma. Mol BioSyst.

[55] Pahlevan Kakhki M, Nikravesh A, Shirvani Farsani Z, Sahraian MA, Behmanesh M (2018). HOTAIR but not ANRIL long non-coding RNA contributes to the pathogenesis of multiple sclerosis. Immunology.

[56] Bian EB, Ma CC, He XJ, Wang C, Zong G, Wang HL (2016). Epigenetic modification of miR-141 regulates SKA2 by an endogenous ‘sponge’ HOTAIR in glioma. Oncotarget..

[57] Ke J, Yao YL, Zheng J, Wang P, Liu YH, Ma J (2015). Knockdown of long non-coding RNA HOTAIR inhibits malignant biological behaviors of human glioma cells via modulation of miR-326. Oncotarget..

[58] Li P, Xu T, Zhou X, Liao L, Pang G, Luo W (2017). Downregulation of miRNA-141 in breast cancer cells is associated with cell migration and invasion: involvement of ANP32E targeting. Cancer Med.

[59] Sun MY, Zhu JY, Zhang CY, Zhang M, Song YN, Rahman K (2017). Autophagy regulated by lncRNA HOTAIR contributes to the cisplatin-induced resistance in endometrial cancer cells. Biotechnol Lett.

[60] Sharma Saha S, Roy Chowdhury R, Mondal NR, Chakravarty B, Chatterjee T, Roy S (2016). Identification of genetic variation in the lncRNA HOTAIR associated with HPV16-related cervical cancer pathogenesis. Cell Oncol.

[61] Ozes AR, Miller DF, Ozes ON, Fang F, Liu Y, Matei D (2016). NF-kappaB-HOTAIR axis links DNA damage response, chemoresistance and cellular senescence in ovarian cancer. Oncogene.

[62] Ding W, Ren J, Ren H, Wang D (2017). Long noncoding RNA HOTAIR modulates MiR-206-mediated Bcl-w signaling to facilitate cell proliferation in breast cancer. Sci Rep.

[63] Yiwei T, Hua H, Hui G, Mao M, Xiang L (2015). HOTAIR interacting with MAPK1 regulates ovarian cancer skov3 cell proliferation, migration, and invasion. Med Sci Monit.

[64] Deng J, Yang M, Jiang R, An N, Wang X, Liu B (2017). Long non-coding RNA HOTAIR regulates the proliferation, self-renewal capacity, tumor formation and migration of the cancer stem-like cell (CSC) subpopulation enriched from breast cancer cells. PLoS ONE.

[65] Ma MZ, Li CX, Zhang Y, Weng MZ, Zhang MD, Qin YY (2014). Long non-coding RNA HOTAIR, a c-Myc activated driver of malignancy, negatively regulates miRNA-130a in gallbladder cancer. Mol Cancer..

[66] Li Y, Wang Z, Shi H, Li H, Li L, Fang R (2016). HBXIP and LSD1 scaffolded by lncRNA hotair mediate transcriptional activation by c-Myc. Cancer Res.

[67] Pan Y, Wang R, Zhang F, Chen Y, Lv Q, Long G (2015). MicroRNA-130a inhibits cell proliferation, invasion and migration in human breast cancer by targeting the RAB5A. Int J Clin Exp Pathol..

[68] Conger AK, Martin EC, Yan TJ, Rhodes LV, Hoang VT, La J (2016). Argonaute 2 expression correlates with a luminal b breast cancer subtype and induces estrogen receptor alpha isoform variation. Noncod RNA.

[69] Ishibashi M, Kogo R, Shibata K, Sawada G, Takahashi Y, Kurashige J (2013). Clinical significance of the expression of long non-coding RNA HOTAIR in primary hepatocellular carcinoma. Oncol Rep.

[70] Gupta RA, Shah N, Wang KC, Kim J, Horlings HM, Wong DJ (2010). Long non-coding RNA HOTAIR reprograms chromatin state to promote cancer metastasis. Nature.

[71] Sorensen KP, Thomassen M, Tan Q, Bak M, Cold S, Burton M (2013). Long non-coding RNA HOTAIR is an independent prognostic marker of metastasis in estrogen receptor-positive primary breast cancer. Breast Cancer Res Treat.

[72] Bhan A, Hussain I, Ansari KI, Kasiri S, Bashyal A, Mandal SS (2013). Antisense transcript long noncoding RNA (lncRNA) HOTAIR is transcriptionally induced by estradiol. J Mol Biol.

[73] Tao S, He H, Chen Q (2015). Estradiol induces HOTAIR levels via GPER-mediated miR-148a inhibition in breast cancer. J Transl Med.

[74] Xue X, Yang YA, Zhang A, Fong KW, Kim J, Song B (2016). LncRNA HOTAIR enhances ER signaling and confers tamoxifen resistance in breast cancer. Oncogene.

[75] Yu Y, Lv F, Liang D, Yang Q, Zhang B, Lin H (2017). HOTAIR may regulate proliferation, apoptosis, migration and invasion of MCF-7 cells through regulating the P53/Akt/JNK signaling pathway. Biomed Pharmacother.

[76] Wang L, Zeng X, Chen S, Ding L, Zhong J, Zhao JC (2013). BRCA1 is a negative modulator of the PRC2 complex. EMBO J.

[77] Wang L, Huang H (2013). EZH2 takes the stage when BRCA1 loses. Cell Cycle.

[78] Zhang H, Cai K, Wang J, Wang X, Cheng K, Shi F (2014). MiR-7, inhibited indirectly by lincRNA HOTAIR, directly inhibits SETDB1 and reverses the EMT of breast cancer stem cells by downregulating the STAT3 pathway. Stem Cells..

[79] Han L, Zhang HC, Li L, Li CX, Di X, Qu X (2018). Downregulation of long noncoding RNA HOTAIR and EZH2 induces apoptosis and inhibits proliferation, invasion, and migration of human breast cancer cells. Cancer Biother Radiopharm.

[80] Li D, Feng J, Wu T, Wang Y, Sun Y, Ren J (2013). Long intergenic noncoding RNA HOTAIR is overexpressed and regulates PTEN methylation in laryngeal squamous cell carcinoma. Am J Pathol.

[81] Su X, Malouf GG, Chen Y, Zhang J, Yao H, Valero V (2014). Comprehensive analysis of long non-coding RNAs in human breast cancer clinical subtypes. Oncotarget..

[82] Liu XH, Sun M, Nie FQ, Ge YB, Zhang EB, Yin DD (2014). Lnc RNA HOTAIR functions as a competing endogenous RNA to regulate HER2 expression by sponging miR-331-3p in gastric cancer. Mol Cancer..

[83] Zhao W, Geng D, Li S, Chen Z, Sun M (2018). LncRNA HOTAIR influences cell growth, migration, invasion, and apoptosis via the miR-20a-5p/HMGA2 axis in breast cancer. Cancer Med.

[84] Ding J, Yeh CR, Sun Y, Lin C, Chou J, Ou Z (2018). Estrogen receptor beta promotes renal cell carcinoma progression via regulating LncRNA HOTAIR-miR-138/200c/204/217 associated CeRNA network. Oncogene.

[85] Piperigkou Z, Bouris P, Onisto M, Franchi M, Kletsas D, Theocharis AD (2016). Estrogen receptor beta modulates breast cancer cells functional properties, signaling and expression of matrix molecules. Matrix Biol.

[86] Ma L, Young J, Prabhala H, Pan E, Mestdagh P, Muth D (2010). miR-9, a MYC/MYCN-activated microRNA, regulates E-cadherin and cancer metastasis. Nat Cell Biol.

[87] Kim HJ, Lee DW, Yim GW, Nam EJ, Kim S, Kim SW (2015). Long non-coding RNA HOTAIR is associated with human cervical cancer progression. Int J Oncol.

[88] Battistelli C, Cicchini C, Santangelo L, Tramontano A, Grassi L, Gonzalez FJ (2017). The Snail repressor recruits EZH2 to specific genomic sites through the enrollment of the lncRNA HOTAIR in epithelial-to-mesenchymal transition. Oncogene.

[89] Xu F, Zhang J (2017). Long non-coding RNA HOTAIR functions as miRNA sponge to promote the epithelial to mesenchymal transition in esophageal cancer. Biomed Pharmacother.

[90] Ding C, Cheng S, Yang Z, Lv Z, Xiao H, Du C (2014). Long non-coding RNA HOTAIR promotes cell migration and invasion via down-regulation of RNA binding motif protein 38 in hepatocellular carcinoma cells. Int J Mol Sci.

[91] Wu J, Zhou XJ, Sun X, Xia TS, Li XX, Shi L (2017). RBM38 is involved in TGF-beta-induced epithelial-to-mesenchymal transition by stabilising zonula occludens-1 mRNA in breast cancer. Br J Cancer.

[92] Zhou Y, Wang C, Liu X, Wu C, Yin H (2017). Long non-coding RNA HOTAIR enhances radioresistance in MDA-MB231 breast cancer cells. Oncol Lett.

[93] Zhou Y, Zhang C, Qin Q, Zhu H, Liu J, Cheng H (2016). Overexpression of long non-coding RNA HOTAIR enhances breast cancer radioresistance via RhoC-Akt pathway by targeting HOXD10. Int J Clin Exp Pathol..

[94] Li Z, Qian J, Li J, Zhu C (2019). Knockdown of lncRNA-HOTAIR downregulates the drug-resistance of breast cancer cells to doxorubicin via the PI3K/AKT/mTOR signaling pathway. Exp Ther Med.

[95] Bhan A, Hussain I, Ansari KI, Bobzean SA, Perrotti LI, Mandal SS (2014). Bisphenol-A and diethylstilbestrol exposure induces the expression of breast cancer associated long noncoding RNA HOTAIR in vitro and in vivo. J Steroid Biochem Mol Biol.

[96] Zhang A, Zhao JC, Kim J, Fong KW, Yang YA, Chakravarti D (2015). LncRNA HOTAIR enhances the androgen-receptor-mediated transcriptional program and drives castration-resistant prostate cancer. Cell Rep.

[97] Yang XD, Xu HT, Xu XH, Ru G, Liu W, Zhu JJ (2016). Knockdown of long non-coding RNA HOTAIR inhibits proliferation and invasiveness and improves radiosensitivity in colorectal cancer. Oncol Rep.

[98] Jiang Y, Li Z, Zheng S, Chen H, Zhao X, Gao W (2016). The long non-coding RNA HOTAIR affects the radiosensitivity of pancreatic ductal adenocarcinoma by regulating the expression of Wnt inhibitory factor 1. Tumour Biol.

[99] Li N, Meng DD, Gao L, Xu Y, Liu PJ, Tian YW (2018). Overexpression of HOTAIR leads to radioresistance of human cervical cancer via promoting HIF-1alpha expression. Radiat Oncol..

[100] Jing L, Yuan W, Ruofan D, Jinjin Y, Haifeng Q (2015). HOTAIR enhanced aggressive biological behaviors and induced radio-resistance via inhibiting p21 in cervical cancer. Tumour Biol.

[101] Yamamoto S, Han W, Kim Y, Du L, Jamshidi N, Huang D (2015). Breast cancer: radiogenomic biomarker reveals associations among dynamic contrast-enhanced MR imaging, long noncoding RNA, and metastasis. Radiology..

[102] Hu X, Ding D, Zhang J, Cui J (2019). Knockdown of lncRNA HOTAIR sensitizes breast cancer cells to ionizing radiation through activating miR-218. Biosci Rep.

[103] Teschendorff AE, Lee S-H, Jones A, Fiegl H, Kalwa M, Wagner W (2015). HOTAIR and its surrogate DNA methylation signature indicate carboplatin resistance in ovarian cancer. Genome Med.

[104] Wang L, Dong P, Wang W, Huang M, Tian B (2017). Gemcitabine treatment causes resistance and malignancy of pancreatic cancer stem-like cells via induction of lncRNA HOTAIR. Exp Ther Med..

[105] Yardley D, Coleman R, Conte P, Cortes J, Brufsky A, Shtivelband M (2018). nab-Paclitaxel plus carboplatin or gemcitabine versus gemcitabine plus carboplatin as first-line treatment of patients with triple-negative metastatic breast cancer: results from the tnAcity trial. Ann Oncol.

[106] Lv R, Zhang J, Zhang W, Huang Y, Wang N, Zhang Q (2018). Circulating HOTAIR expression predicts the clinical response to neoadjuvant chemotherapy in patients with breast cancer. Cancer Biomark..

[107] Yan J, Dang Y, Liu S, Zhang Y, Zhang G (2016). LncRNA HOTAIR promotes cisplatin resistance in gastric cancer by targeting miR-126 to activate the PI3K/AKT/MRP1 genes. Tumour Biol.

[108] Liu Z, Sun M, Lu K, Liu J, Zhang M, Wu W (2013). The long noncoding RNA HOTAIR contributes to cisplatin resistance of human lung adenocarcinoma cells via downregualtion of p21(WAF1/CIP1) expression. PloS ONE..

[109] Fang S, Gao H, Tong Y, Yang J, Tang R, Niu Y (2016). Long noncoding RNA-HOTAIR affects chemoresistance by regulating HOXA1 methylation in small cell lung cancer cells. Lab Investig..

[110] Wang Y, Wang H, Song T, Zou Y, Jiang J, Fang L (2015). HOTAIR is a potential target for the treatment of cisplatinresistant ovarian cancer. Mol Med Rep.

[111] Li J, Yang S, Su N, Wang Y, Yu J, Qiu H (2016). Overexpression of long non-coding RNA HOTAIR leads to chemoresistance by activating the Wnt/beta-catenin pathway in human ovarian cancer. Tumour Biol.

[112] Xiao Z, Qu Z, Chen Z, Fang Z, Zhou K, Huang Z (2018). LncRNA HOTAIR is a prognostic biomarker for the proliferation and chemoresistance of colorectal cancer via MiR-203a-3p-mediated Wnt/ss-catenin signaling pathway. Cell Physiol Biochem.

[113] Li P, Zhang X, Wang L, Du L, Yang Y, Liu T (2017). lncRNA HOTAIR contributes to 5FU resistance through suppressing miR-218 and activating NF-kappaB/TS signaling in colorectal cancer. Mol Ther Nucleic Acids..

[114] Dijkgraaf EM, Heusinkveld M, Tummers B, Vogelpoel LT, Goedemans R, Jha V (2013). Chemotherapy alters monocyte differentiation to favor generation of cancer-supporting M2 macrophages in the tumor microenvironment. Cancer Res.

[115] Teschendorff AE, Lee SH, Jones A, Fiegl H, Kalwa M, Wagner W (2015). HOTAIR and its surrogate DNA methylation signature indicate carboplatin resistance in ovarian cancer. Genome Med..

[116] Chen J, Lin C, Yong W, Ye Y, Huang Z (2015). Calycosin and genistein induce apoptosis by inactivation of HOTAIR/p-Akt signaling pathway in human breast cancer MCF-7 cells. Cell Physiol Biochem.

[117] Chiyomaru T, Fukuhara S, Saini S, Majid S, Deng G, Shahryari V (2014). Long non-coding RNA HOTAIR is targeted and regulated by miR-141 in human cancer cells. J Biol Chem..

[118] Wang YL, Overstreet AM, Chen MS, Wang J, Zhao HJ, Ho PC (2015). Combined inhibition of EGFR and c-ABL suppresses the growth of triple-negative breast cancer growth through inhibition of HOTAIR. Oncotarget..

[119] Ozes AR, Wang Y, Zong X, Fang F, Pilrose J, Nephew KP (2017). Therapeutic targeting using tumor specific peptides inhibits long non-coding RNA HOTAIR activity in ovarian and breast cancer. Sci Rep.

